# Image Quality Assessment Tool for Conventional and Dynamic Magnetic Resonance Imaging Acquisitions

**DOI:** 10.3390/jimaging10050115

**Published:** 2024-05-09

**Authors:** Katerina Nikiforaki, Ioannis Karatzanis, Aikaterini Dovrou, Maciej Bobowicz, Katarzyna Gwozdziewicz, Oliver Díaz, Manolis Tsiknakis, Dimitrios I. Fotiadis, Karim Lekadir, Kostas Marias

**Affiliations:** 1 Computational BioMedicine Laboratory, Institute of Computer Science, Foundation for Research and Technology—Hellas (FORTH), 70013 Heraklion, Greece; karatzan@ics.forth.gr (I.K.); dovrou@ics.forth.gr (A.D.); tsiknaki@ics.forth.gr (M.T.); kmarias@ics.forth.gr (K.M.); 2 School of Medicine, University of Crete, 71003 Heraklion, Greece; 3 2nd Department of Radiology, Medical University of Gdansk, 80-214 Gdansk, Poland; mbobowicz@gumed.edu.pl (M.B.); katarzyna.gwozdziewicz@gumed.edu.pl (K.G.); 4 Departament de Matemàtiques i Informàtica, Universitat de Barcelona, 08007 Barcelona, Spain; oliver.diaz@ub.edu (O.D.); karim.lekadir@ub.edu (K.L.); 5 Computer Vision Center, 08193 Bellaterra, Spain; 6 Department of Electrical and Computer Engineering, Hellenic Mediterranean University, 71410 Heraklion, Greece; 7 Biomedical Research Institute, Foundation for Research and Technology—Hellas (FORTH), 45500 Ioannina, Greece; fotiadis@uoi.gr; 8 Institució Catalana de Recerca i Estudis Avançats (ICREA), 08010 Barcelona, Spain

**Keywords:** magnetic resonance imaging (MRI), image quality, tool, artifacts, noise, contrast, full-reference, no-reference, signal-to-noise ratio (SNR), contrast-to-noise ratio (CNR), breast MRI

## Abstract

Image quality assessment of magnetic resonance imaging (MRI) data is an important factor not only for conventional diagnosis and protocol optimization but also for fairness, trustworthiness, and robustness of artificial intelligence (AI) applications, especially on large heterogeneous datasets. Information on image quality in multi-centric studies is important to complement the contribution profile from each data node along with quantity information, especially when large variability is expected, and certain acceptance criteria apply. The main goal of this work was to present a tool enabling users to assess image quality based on both subjective criteria as well as objective image quality metrics used to support the decision on image quality based on evidence. The evaluation can be performed on both conventional and dynamic MRI acquisition protocols, while the latter is also checked longitudinally across dynamic series. The assessment provides an overall image quality score and information on the types of artifacts and degrading factors as well as a number of objective metrics for automated evaluation across series (BRISQUE score, Total Variation, PSNR, SSIM, FSIM, MS-SSIM). Moreover, the user can define specific regions of interest (ROIs) to calculate the regional signal-to-noise ratio (SNR) and contrast-to-noise ratio (CNR), thus individualizing the quality output to specific use cases, such as tissue-specific contrast or regional noise quantification.

## 1. Introduction

Image quality in the medical domain is a key concept for diagnostic image interpretation. Image quality is especially important when assessed by human experts as a measure of image diagnostic value and the ability to present disease manifestation and identify subtle differences among pathology or physiology entities. However, it is equally important when images are used as input in artificial intelligence (AI) algorithms for achieving adequate performance on segmentation or classification tasks. Image quality information is an important factor not only for AI model explainability but also for assessing fairness and robustness when images acquired from different sites, vendors, or imaging protocols are considered [1]. This is extremely important, as current AI needs to promote the aggregation of large data volumes in image repositories, such as the “The Cancer Imaging Archive” (TCIA) [2] repository or EUCAIM initiative [3], where many clinical sites are invited and encouraged to contribute data that show high variability in their acquisition settings.

The notion of image quality is more complex where medical images are concerned, as it is related to the ability to deliver an accurate and specific clinical outcome. Moreover, diagnostic quality may be outlined by different attributes or weaknesses depending on the recipient of the image, if it is a diagnostic decision support system or the field expert using human perception, even for the same clinical question. Objective image quality metrics are also used to provide an alternative to manual subjective scoring, avoiding issues of inter- or intra-observer variability. These metrics evaluate image quality based on expected image statistics. Ideally, a valuable quality metric correlates well with the subjective perception of quality by a human rater. The selection of objective metrics that correlate with the evaluation based on human perception is still an open field of research [4,5,6,7,8].

Assessing image quality can contribute to efficiently building large databases, as it is relevant to a number of different aspects that AI repositories value. It serves for image harmonization, as it can highlight images requiring post-processing actions such as noise reduction or motion correction. Image Quality Assessment (IQA) supports fairness [9] by assessing sufficiency among different scanners, sites, or other conditions. It holds an important role in explainability [10], as data perturbations with regard to quality may be relevant to consistency in results and transferability of the outcome to different sites. Moreover, it can support the evaluation of real-world clinical usability by applying exclusion criteria and linking them to specific image attributes, such as local or global signal-to-noise ratio (SNR), pixel size, or the presence of artifacts. It can also be a part of robustness evaluation, as data quality can serve to produce group-specific studies, either by exclusively eliminating image groups by thresholding or by creating mixed groups of desired percentages of quality levels. Moreover, local measurements can assist explanations focusing on specific regions of the image when local degradation factors are observed, such as variable signal reception sensitivity from different radiofrequency coils or coil configurations. 

To date, the human expert’s opinion remains the gold standard for assessing the quality of medical images, as they are the individuals responsible for delivering and signing the final diagnosis. Subsequently, AI-based applications for assessing image quality are oriented and evaluated according to their ability to mimic human opinion. This work presents a software tool enabling a combined evaluation of the quality of a medical image by human experts but also providing a number of automatically calculated objective criteria to support the evaluation, in a single session. 

## 2. Material and Methods

The IQA tool proposed in this work is oriented to explore the sequences and register degrading factors that are most relevant to the current practice of multi-parametric (mp) breast magnetic resonance imaging (MRI) examinations in the frame of an EU-funded project (https://radioval.eu/, accessed on 5 May 2023). A typical mp breast MRI protocol consists of a number of conventional (T1- or T2-weighted images) in axial or sagittal orientation and dynamic T1-weighted acquisitions (Dynamic Contrast Enhanced, DCE) with approximately 90 sec temporal resolution for a number of time points (3 to 6), usually at identical orientation as the conventional imaging. Diffusion Weighted Imaging (DWI) is also part of an mp breast MRI protocol.

### 2.1. Working Environment

The tool can work as a plugin within the freely available imaging software Mango V4.1 (https://mangoviewer.com/, accessed on 1 May 2024) or can work as a stand-alone tool, as a windowed application. The application can also be executed via the command line provided the appropriate arguments, although it will launch its Graphical User Interface (GUI) once the analysis is completed. The advantage of integrating into Mango’s environment is the ability to use the additional functionalities related to viewing or performing some post-processing applications. The latter is related to faster and more efficient delineation of user-defined regions of interest (ROIs), which are used to perform local quality measurements. However, this action is optional and is not a prerequisite for the successful completion of the evaluation task.

The window application of the tool is a comprehensive User Interface (UI) image quality app for conventional and dynamic MR series to report image quality and types of artifacts. The Tkinter package (the standard Python interface to the Tcl/Tk GUI toolkit) was used to build the application’s UI. The ttkbootstrap (1.10.×) theme extension for tkinter was also used to provide a modern flat-style theme inspired by the widely used front-end toolkit Bootstrap. The image quality metrics, which are the No-Reference (NR) and Full-Reference (FR) metrics, were calculated using the PyTorch Image Quality (PIQ) library (version 0.8.0) [11]. Furthermore, fundamental Python libraries for data manipulation and mathematical calculations were used, such as numpy (v1.26.0), pandas (v2.1.3), pydicom (v2.4.3), matplotlib (v3.8.1), and pytorch (v2.1.1).

### 2.2. Input

The tool supports a prevalent file format in radiology, DICOM (.dcm), though it could be extended with ease to support other medical imaging file formats in a future version, i.e., NifTI (.nii) or ITK MetaImages (.mha). The tool is provided with a couple of inputs: the type of images to be utilized (DCE or Conventional), which serves for initialization and configuration purposes and defines the processing that will be performed, and the directory path to the medical images. The tool iteratively parses either a single directory input (containing DICOM files) or resources through a directory of directories, to extract the image volumes and the respective image metadata.

### 2.3. IQA Tool Components

The full functionality of the IQA tool includes the presentation of descriptive attributes for the scanner/sequence, the submission of the subjective metrics, as well as the presentation of objective image metrics (NR metrics for conventional images, NR and FR metrics for dynamic studies, and statistical metrics for any type of image concerning relative or local measurements in user-defined regions) (Table 1).

Full-Reference metrics are measurements that compare the quality of a target image to the quality of a reference image. The FR metrics deployed in the tool are Peak SNR (PSNR), Structural Similarity Index Measure (SSIM) [12], Multi-Scale Structural Similarity Index Measure (MS-SSIM) [13], and Feature Similarity Index Measure (FSIM) [14]. The PSNR metric is defined as the ratio between the maximum possible power of a signal and the power of corrupting noise. The SSIM is a method to predict image quality by focusing on structural distortions, measuring the structural similarity between two images. Luminance, contrast, and structural similarity components are calculated automatically from the image and then combined into a single quality map. The MS-SSIM metric, the Multi-scale SSIM, is an extension of SSIM and measures SSIM on five different scales, computing contrast and similarity on all levels while measuring luminance only at the final scale. The FSIM metric is a method that measures the feature similarity between two images. For all SSIM, MS-SSIM, and FSIM, a value of one indicates perfect similarity between the images compared. They can be used to compare the target image and the reference image directly. In this application, the FR metrics are calculated only for the dynamic series, in which the pre-contrast image is used as the reference image and each consecutive timepoint of the dynamic acquisition as the target image. These metrics are used to identify motion between identical acquisitions in dynamic series, as a local decrease in the index. The PSNR metric can be used as a measure of global SNR, but PSNR is also indicative of the correct timing of contrast administration.

No-Reference metrics compute quality scores based on expected image statistics. Metrics used in this implementation are the BRISQUE score [15] and Total Variation, which are applied to any image series. Total Variation shows the integral of the absolute image gradient. High values of Total Variation indicate a high image gradient and thus are likely to identify images with higher contrast. BRISQUE score is computed using a support vector regression (SVR) model trained on an image database [16] with corresponding differential mean opinion score (DMOS) values that represent the subjective quality of the image. The database contains natural images with known distortion such as compression artifacts, blurring, and noise, and it contains pristine versions of the distorted images. The image to be scored must have at least one of the distortions for which the model was trained. A smaller score of BRISQUE is better, indicating a higher quality of the image.

### 2.4. Output

Outputs, in the form of graphs or text files, are stored in the “IQA” folder within the same directory as the inspected images. This folder contains questionnaire responses in text format, along with image metadata in an Excel file.

### 2.5. Dataset for Module Demonstration

The publicly available Duke Breast Cancer MRI dataset with breast data was used [17,18] to perform basic testing of the tool. The results from the experiments performed on the images of an indicative patient from the dataset with an ID equal to Breast_MRI_056 are presented.

### 2.6. Metrics Evaluation

The NR and the FR metrics were calculated under different conditions in order to evaluate the potential of these objective image metrics in assessing the quality of medical images. More specifically, the NR metrics were calculated for three different slices (indicatively slices 98, 99, and 113) of the same image of the first post-contrast phase to measure the effect of slight in-plane (z-axis, head–foot) patient movement. The three slices of the same image were selected to simulate slightly different image positions for conventional series. The NR metrics were also calculated for the same slices across time phases to investigate the change in the NR metrics when no patient movement is simulated. As another step for testing the tool’s ability to highlight images of degraded quality in a dynamic acquisition, the FR metrics were calculated for identical slices across time passes (no patient motion), and after simulating through-plane patient translocation by disaligning the different time phase series (patient motion during acquisition). To this end, the images of these three slices from the first post-contrast phase were used as input and reference images for the calculation of the FR metrics, expecting to verify the maximum output value in similarity indices. The image of Slice 98 was compared to the image of Slice 98 (identical), Slice 99 (close to the image), and Slice 113 (further in the volume). This comparison could simulate the differences in the images when patient movement is present. Moreover, Slice 98 of the first post-contrast phase was blurred using a Gaussian blurring filter with a kernel size equal to 7 × 7 to further simulate though-plane (transversal) patient motion. The image of the same slice of the second post-contrast phase was used as a reference image, while the original and the blurred version of the image of this slice were used as target images to calculate the FR metrics. To further assess whether the FR metrics can detect the degradation in the image quality due to the blurring effect, the FR metrics were calculated using the original pre-contrast phase image as reference image and original and blurred versions of the images of subsequent phases as target images. More precisely, Gaussian blurring was performed on the third and fifth phases out of the seven phases of a DCE image. The FR metrics were calculated between the same pre-contrast phase image (reference image) and all the subsequent phases with and without blurring (target images).

## 3. Results

### 3.1. Frontend and Visualization

Image Quality Assessment’s frontend consists of five distinct components: 1. working directory (windowed application) or image viewer (Mango Viewer v4.1) [19], 2. descriptive attributes, 3. questionnaire, 4. graphical representation of objective image quality metrics, and 5. the IQA plugin for the Mango viewer.

#### 3.1.1. First Component—The Working Directory

**Figure 1:**
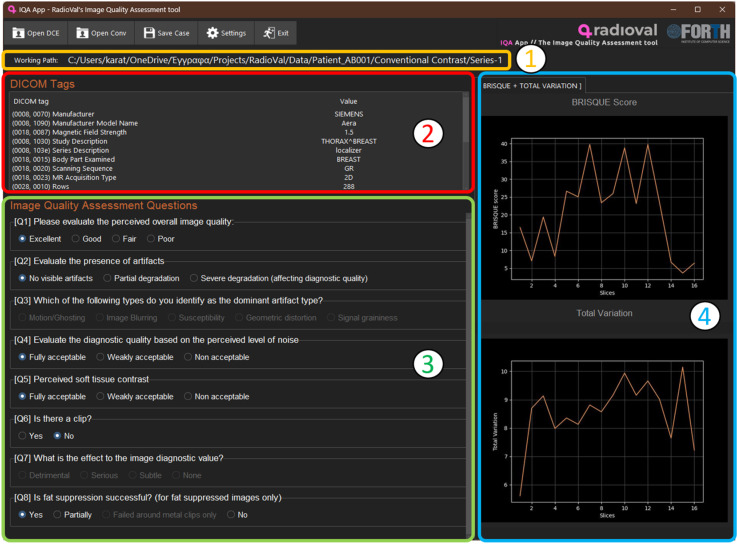
Windowed application showing the distinct compartments: 1: working path, 2: DICOM tags extracted from the sequence headers, 3: questionnaire to be addressed by the expert (only this part is mandatory for completing the IQA evaluation), 4: objective quality metrics for the total number of slices in the selected sequence. The windowed application does not support ROI-based SNR and CNR measurements.

The first component of the tool displays the path of the current working directory (as shown in Figure 1 in a yellow box). This directory contains the currently used data and serves as the primary location for file operations and data processing within the application.

#### 3.1.2. Second Component—Descriptive Attributes

The second component (red box in Figure 1) presents informative values extracted from the images’ DICOM header and aims to offer a rough outline of the sequence parameters related to image quality. No user action is necessary here, as these attributes serve a purely informative purpose.

#### 3.1.3. Third Component—The Questionnaire

The third component (green box in Figure 1) consists of a concise questionnaire designed to draw users’ attention towards specific attributes often associated with image degradation. The questionnaire starts with the overall impression of the expert, regardless of the specific reasons for degradation, if present. Unless scored as excellent, the user is guided to analyze the specific aspects of the image, related to contrast, noise, and artifacts. It aims to assist users in breaking down their overall perception of image quality into individual elements. This breakdown helps identify the factors contributing to any suboptimal results. It must be noted that this is the only part of the task that is obligatory, as the requested information is recorded here. It can therefore be used as a standalone application and is adequate to complete the evaluation task without the need for the user to inspect the graphs of the objective metrics.

#### 3.1.4. Fourth Component—Graphical Representation of Objective Metrics

The fourth component (blue box in Figure 1) consists of graphical depictions of objective image quality metrics, aiding clinicians in obtaining quantifiable evidence to support their decisions. No-Reference metrics are computed per slice for all images, while Full-Reference metrics are computed for 10 equidistant slice locations across all time points. For each selected slice, each FR metric is calculated using the image slice at each timepoint as a target image and the same image slice at the pre-contrast phase as the reference image. Moreover, as they are measured and presented across slices or time points, they serve to provide a time-efficient application, as the local or global minima easily identified on the graph attract the user’s attention to the slices or time points most suspicious for degradation.

#### 3.1.5. Fifth Component—The IQA Plugins for Mango

**Figure 2:**
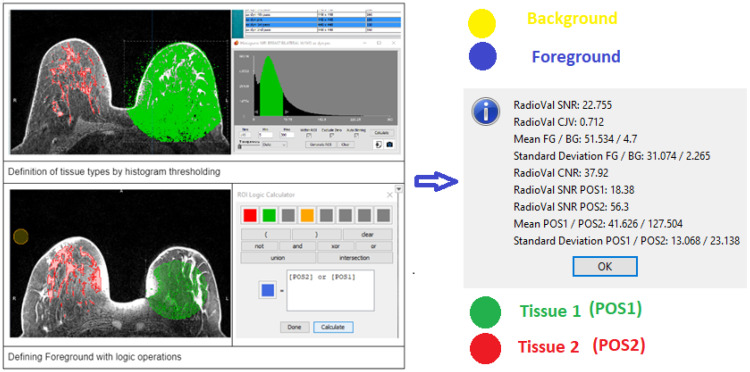
Workflow for measurement of ROI-based metrics (segmentation based on histogram sub-sections, ROI logical operations for defining the foreground ROI) related to tissue-specific or local SNR and CNR.

The fifth component is a set of plugins for the Mango DICOM viewer (available only in the Mango-supported version and not in the stand-alone application), where the user can browse the whole series of images for evaluation. Basic functionalities such as windowing, zooming, scrolling through slices or time points, or changing the orientation are available through Mango. There are also tools available for performing mathematical calculations among images, importing, exporting, loading ROIs, or viewing the image histogram (Figure 2).

The IQA component is imported as a .jar file from the main Mango menu and is then available through the plugin list on the image menu. The IQA’s dedicated plugins facilitate the local assessment of SNR or CNR or relative evaluation of those metrics, i.e., between two different tissue types or between the foreground and the background. The user has to define ROIs as either indicative or partially or totally occupying the area of interest and name them with specified names (pos1,2 for tissue types 1, 2, FG, and BG for foreground and background, respectively). For all available labels, several statistical metrics are measured. When the plugin is launched, the tool calculates the mean and standard deviation of each region. The derived indices from the calculation are SNR, coefficient of variation of the foreground patch for shading artifacts (CVP), coefficient of joint variation (CJV) [20], and relative SNR [21], presenting the contrast and noise indices of the images numerically. To facilitate the process, an additional plugin can initialize the four (4) ROIs and name them. The user has to define a spherical radius and then drag and drop them in the corresponding areas. The ROIs initiated by the plugin can be modified by Mango’s toolkit.

### 3.2. Tool Testing

**Figure 3:**
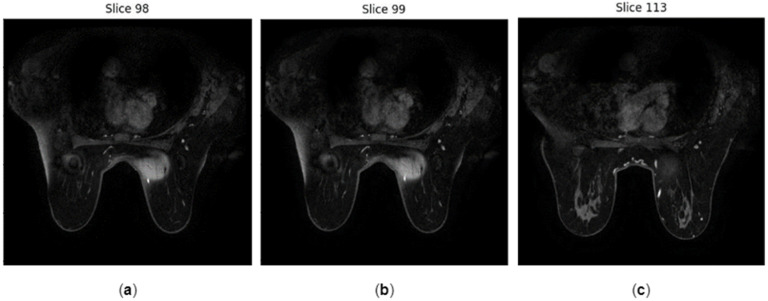
Images of different slices of the first post-contrast phase of a patient with ID Breast_MRI_056: (**a**) Slice 98; (**b**) Slice 99; (**c**) Slice 113. Comparison among different slice locations serves to simulate patient movement in the z-axis during dynamic acquisitions for performing feasibility testing of in-plane motion detection.

**Figure 4:**
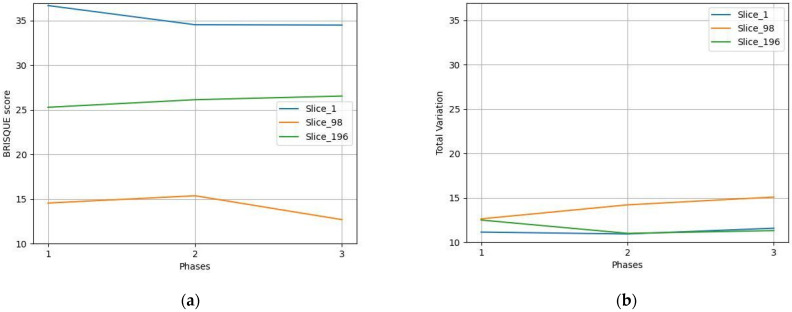
No-Reference metrics for three different slices of a patient with ID Breast_MRI_056 in three post-contrast phases: (**a**) BRISQUE score for each slice in each post-contrast phase; (**b**) total variation for each slice in each post-contrast phase.

**Figure 5:**
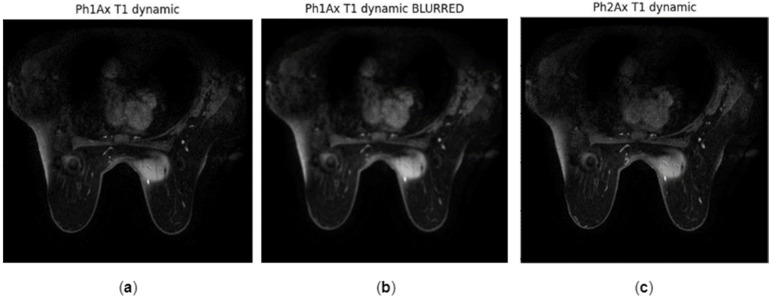
Images of a patient with ID Breast_MRI_056: (**a**) Slice 98 of the first post-contrast phase; (**b**) Slice 98 of the first post-contrast phase after applying a Gaussian blurring filter with a kernel size equal to 7 × 7; (**c**) Slice 98 of the second post-contrast phase. Applying blurring serves to simulate patient movement in the x-y plane for performing feasibility testing of through-plane motion detection.

**Figure 6:**
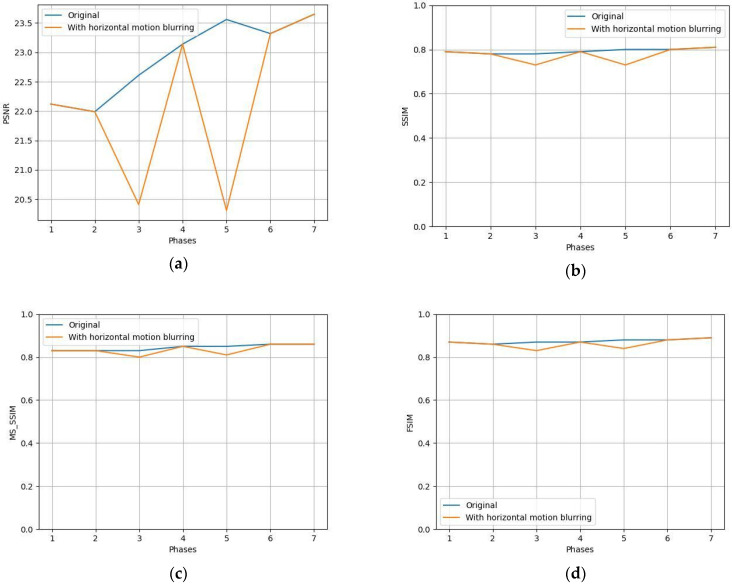
Feasibility testing of identifying degraded time points inside a time-series dynamic acquisition. Full-Reference metrics were calculated for all time phases for the original dataset, as well as the original dataset after applying blurring filters in time phases 3 and 5 out of 7. (**a**) Peak Signal-to-Noise Ratio (PSNR); (**b**) Structural Similarity Index Measure (SSIM); (**c**) Multi-Scale Structural Similarity Index Measure (MS-SSIM); (**d**) Feature Similarity Index Measure (FSIM).

Several tests were conducted under specific hypothetical scenarios as feasibility testing of the ability of the tool to support the expert in identifying the most suspicious slices of compromised quality in a time-efficient manner. The NR metrics of three indicative slices (Slice 98, 99, and 113 shown in Figure 3) were calculated to compare the quality of slightly different image positions, as shown in Table 2. Both NR metrics agree and indicate that Slice 113 is of slightly better quality since BRISQUE score and Total Variation have the smallest and largest values, respectively. Furthermore, the NR metrics calculated on images of the same patient indicate that the median slice (Slice 98) is of better quality than the slices at the beginning and the end of the sequence (Figure 4), since smallest values of BRISQUE score and largest values of Total Variation are observed for the median slice across all time phases. However, a small variation in the values of the NR metrics of the same slice across the three time phases was observed, confirming that the quality of the different series of a DCE image is expected to be similar across the time phases. Furthermore, these slices of the first post-contrast phase were compared to assess the FR metrics (Table 3). The results verified the potential of the FR metrics to assess images when patient movement is present. The values of the structure-based metrics were equal to 1 when identical images (i.e., Slice 98) were compared. As expected for the hypothesis of the testing, the values of the structure-based metrics were close to 1, when consecutive images of the same series were compared (i.e., Slice 98 and Slice 99). When comparing distant images with different anatomy (i.e., Slice 98 and Slice 113), the values of these metrics drop significantly, indicating the discrepancy between these images. Finally, phase 1 post-contrast was blurred using a Gaussian blurring filter with a kernel size equal to 7 × 7 to simulate through-plane (transversal) patient motion (Figure 5), and the differences in the resulting metrics are shown in Table 4. The values of the FR metrics slightly drop when comparing the blurred version of the image with the reference second post-contrast phase image. In the testing phase of the tool with data from the project, and feasibility testing for feedback locally at the clinical sites, the same experiment was conducted with and without blurring in phases three and five out of seven, as shown in Figure 6. The values of the FR metrics between the pre-contrast phase image (reference image) and the subsequent seven phases (target images) are indicated with the blue line. The values of the FR metrics between the pre-contrast phase image (reference image) and the subsequent seven phases after applying blurring in the third and fifth phases (target images) are depicted with the orange line. The values of all FR metrics drop when comparing the blurred version of the images in phases three and five with the same reference image, indicating image quality degradation in these phases due to the blurring effect.

## 4. Discussion

The IQA tool presented in this work has dedicated components for sequence evaluation with respect to the parameters, a means to register a number of user-defined image quality attributes, as well as provides quantitative objective metrics for the support of the user. Testing the ability of the tool to identify slices most suspicious for image degradation has been conducted as feasibility proof to provide a time-efficient working environment to highlight the slices or sequences most affected by degradation factors. The feasibility testing comprised a comparison among metrics when patient motion was simulated in-plane and through-plane of acquisition by applying blurring filters and translocating slices across time phases, respectively.

Regarding conventional diagnostics, it can be used to assess the ability to visualize, identify disease manifestation signs, or compare images acquired under different protocol setups both visually and numerically under certain circumstances. In the frame of a multi-centric or variable protocol study, it can help optimize protocol parameters under real clinical situations rather than resorting to the use of test objects, which cannot capture disease heterogeneity, the effect of artifacts, and human behavior. For AI methodologies, the proposed tool could serve to enhance robustness and explainability as well as promote the fairness of AI algorithms. Such actions are in the core interest of AI professionals and the medical community, as they are essential to building confidence and trust and being adopted in everyday clinical practice.

The only mandatory part of the evaluation is the human-based evaluation, performed by the completion of a questionnaire by the user. The automatic objective metrics calculation and the manual ROI-based analysis are the optional features supporting the user in defending his/her evaluation. Since the tool was designed to meet the needs of the RadioVal project for IQA, specific characteristics related to mp MRI of the breast are examined in the current version of the tool’s questionnaire, i.e., the ability to suppress fat, the presence and degrading effect of metal clips, dynamic acquisitions, etc. However, the tool can be applied to other body parts or imaging modalities, as it can be configured by selecting the appropriate parts relevant to other use cases. Conventional series of any contrast and in any orientation are assessed by human experts and NR objective metrics, while dynamic series are assessed by human experts and FR objective metrics, taking as reference the pre-contrast phase. The tool can be used as a stand-alone application or can be hosted as a plugin in a DICOM viewer. The latter option enables local assessment of different image sub-parts by calculating medically oriented objective metrics in user-defined image areas. Local assessment of relative SNR and contrast-to-noise ratio (CNR) metrics can also be applied to measure tissue-specific evaluation or assessment in different sub-parts of the image.

Each attribute of the tool serves a specific purpose. The presentation of selected attributes related to quality derived from the DICOM header can be used to verify that the perceived image characteristics correlate with the objective attributes of the sequence. For example, a slightly blurred image can have a low score based on the expert’s evaluation. However, identifying the etiology of blurring is feasible once the sequence characteristics are taken into account. Thus, patient- or protocol-related blurring is evident. An image of low resolution, as revealed by sub-optimal characteristics in the “number of frequency/phase encoding steps”, can be differentiated from an image compromised by patient involuntary motion during acquisition, even though the image parameters are tailored for excellent image quality. Moreover, an image having a high SNR can score differently once a slice thickness above the diagnostic threshold is chosen.

The tool registers the human experts’ opinions by addressing several questions related to image-degrading factors. They are also asked to provide an overall score of the perceived image quality. Each question aims to draw attention to one specific aspect of the image to decompose the overall impression into specific etiologies of degradation (contrast, noise, or artifacts) to provide a concise but comprehensive image profile.

Graphical representation of objective metrics across series or time points serves to provide a time-efficient experience as an alert for selectively inspecting the group of images where metrics present a decrease. It has to be noted that a single time point of a dynamic series can be considered a conventional series when used in isolation from the rest of the time phases and is therefore assessed by NR metrics. A local or global minimum value can suggest a selective evaluation of those particular slices scoring low in one or both objective evaluations by the user, as being hypothetically the worst representations of the anatomy.

The use of ROI-based local evaluation of SNR and CNR statistical metrics has been added, as none of the deployed FR or NR metrics is medically oriented, and it can be possible either to find discordance among them for the scoring of image quality, or it is possible that they fail to disassociate diagnostic value from image quality. Such examples can be ignoring areas of imperfect excitation appearing black, variable coil sensitivity, or, on the contrary, the case of a persistent non-avoidable artifact not covering the area of diagnostic interest.

Similar implementations have been developed, with the vast majority focusing on brain MRI studies (MRIQC [22], Qoala-T [23], MRQy [24]). Compared to the existing software solutions for MRI IQA, the main advantages of the tool presented herein are the simultaneous subjective and objective evaluation, the ability to assess images independently of the anatomical site (also possible in [24]), and the ability to perform local tissue-specific calculations of medically oriented metrics. The flexibility of localized measures widely accepted for medical image evaluation (ROI-based statistical metrics of mean and standard deviation for specific sub-regions) has not been part of any IQA tool currently available.

Additionally, the integration of subjective and objective evaluation for an image at a single session is an important attribute of the tool. The automatic extraction of a number of objective metrics alongside the experts’ opinions on various aspects of possible image degradation can be a very powerful starting point to create associations between the most powerful metrics to capture the users’ opinion, which remains the holy grail. Moreover, it can highlight the correlation of specific artifacts or degrading factors to the most sensitive objective markers for each factor, apart from the overall image evaluation.

Limitations concerning this version of the tool are as follows. (1) The ability to support image formats other than DICOM. Future work will be able to support more imaging formats, such as NifTI or ITK MetaImages (.mha); however, this will provide incomplete information concerning the DICOM header tags that are an important part of the rationale of this work. (2) Moreover, the current version of the application is not tailored to the evaluation of other 4D datasets such as DWI, which are very important for diagnosis. They present specific challenges regarding IQA, as artifacts (geometric distortion) and SNR drops on high b values are expected.

Both of the aforementioned limitations are considered, and the tool can accommodate changes to address the need for more imaging formats, as well as DWI sequences. Future actions will include further testing in clinical environments, and the tool is expected to be optimized by experts’ feedback in the frame of the RadioVal project and beyond.

## 5. Conclusions

The combined registration of human perception and objective metrics regarding image quality at a single session for identical images is the strength of the presented tool, alongside time efficiency and the addition of medically oriented ROI-specific metrics for evidence-based support of the outcome. Such a tool is important to rate data according to different aspects of quality and to divide data sub-groups depending on either the degree or etiology of degradation. Therefore, it registers important information that can be combined with the diagnostic value of a certain MRI set-up, scanner update, hardware changes, or protocol optimization actions, and it supports the analysis of robustness, fairness, and explainability of AI models on the basis of interpreting the effect of image quality on the outcome. Moreover, capturing both subjective evaluation and objective metrics can be used to produce correlations between human-perceived degrading factors and metrics calculated by computers for variable research or clinical use cases.

## Figures and Tables

**Table 1:** No-Reference and Full-Reference metrics presented in the IQA tool.

	Image Quality Metrics
No-Reference metrics	BRISQUE score
	Total Variation
Full-Reference metrics	Peak Signal-to-Noise Ratio (PSNR)
	Structural Similarity Index Measure (SSIM)
	Multi-Scale Structural Similarity Index Measure (MS-SSIM)
	Feature Similarity Index Measure (FSIM)

**Table 2:** No-Reference metrics for the examined slices (Slice 98, 99, and 113).

NR Metrics	Slice 98	Slice 99	Slice 113
BRISQUE	13.51	13.29	11.27
Total Variation	13.39	14.13	15.76

**Table 3:** Full-Reference metrics for Slice 98 (target) compared to Slice 98, 99, and 113 (references), respectively, of the same series.

FR Metrics	Reference Image		
	**Slice 98**	**Slice 99**	**Slice 113**
Peak Signal-to-Noise Ratio (PSNR)	80.0	32.88	22.93
Structural Similarity Index Measure (SSIM)	1.0	0.90	0.59
Multi-Scale Structural Similarity Index Measure (MS-SSIM)	1.0	0.95	0.62
Feature Similarity Index Measure (FSIM)	1.0	0.93	0.76

**Table 4:** Full-Reference metrics for the image of the first post-contrast phase (original and blurred) compared to the second post-contrast phase. The reference image is the second post-contrast phase.

FR Metrics	Input Image	
	Phase 1	Phase 1 Blurred
PSNR	29.17 ± 2.07	26.66 ± 2.38
SSIM	0.83 ± 0.03	0.82 ± 0.03
MS-SSIM	0.90 ± 0.02	0.89 ± 0.02
FSIM	0.92 ± 0.02	0.92 ± 0.02

## Data Availability

The software tool testing has been based only on public datasets (https://sites.duke.edu/mazurowski/resources/breast-cancer-mri-dataset/, accessed on 29 April 2023).
